# *Piriformospora indica* alleviates soda saline-alkaline stress in *Glycine max* by modulating plant metabolism

**DOI:** 10.3389/fpls.2024.1406542

**Published:** 2024-08-20

**Authors:** Siyu Zhu, Feng Shi, Honghe Li, Yiwen Ding, Wei Chang, Yuan Ping, Fuqiang Song

**Affiliations:** Engineering Research Center of Agricultural Microbiology Technology, Ministry of Education and Heilongjiang Provincial Key Laboratory of Ecological Restoration and Resource Utilization for Cold Region and Key Laboratory of Microbiology, College of Heilongjiang Province and School of Life Sciences, Heilongjiang University, Harbin, China

**Keywords:** natural soda saline-alkaline soil, *Piriformospora indica*, *Glycine max*, biomass, antioxidant defense, photosynthetic gas exchange parameters

## Abstract

Soil salinization is one of the major factors limiting agricultural production. Utilizing beneficial microorganisms like *Piriformospora indica* (*P. indica*) to enhance plant tolerance to abiotic stresses is a highly effective method, but the influence of *P. indica* on the growth of soybean in natural saline-alkaline soil remains unclear. Therefore, we investigated the effects of non-inoculation, *P. indica* inoculation, and fertilization on the growth, antioxidant defense, osmotic adjustment, and photosynthetic gas exchange parameters of soybean under two different levels of saline-alkaline stress in non-sterilized natural saline-alkaline soil. The study found that: 1) *P. indica* inoculation significantly promoted soybean growth, increasing plant height, root length, and biomass. Under mildly saline-alkaline stress, the increases were 11.5%, 16.0%, and 14.8%, respectively, compared to non-inoculated treatment. Under higher stress, *P. indica* inoculation achieved the same level of biomass increase as fertilization, while fertilization only significantly improved stem diameter. 2) Under saline-alkaline stress, *P. indica* inoculation significantly increased antioxidant enzyme activities and reduced malondialdehyde (MDA) content. Under mildly stress, MDA content was reduced by 47.1% and 43.3% compared to non-inoculated and fertilized treatments, respectively. Under moderate stress, the MDA content in the inoculated group was reduced by 29.9% and 36.6% compared to non-inoculated and fertilized treatments, respectively. Fertilization only had a positive effect on peroxidase (POD) activity. 3) *P. indica* inoculation induced plants to produce more osmotic adjustment substances. Under mildly stress, proline, soluble sugars, and soluble proteins were increased by 345.7%, 104.4%, and 6.9%, respectively, compared to non-inoculated treatment. Under higher stress, the increases were 75.4%, 179.7%, and 12.6%, respectively. Fertilization had no significant positive effect on proline content. 4) With increasing stress, soybean photosynthetic capacity in the *P. indica*-inoculated treatment was significantly higher than in the non-inoculated treatment, with net photosynthetic rate increased by 14.8% and 37.0% under different stress levels. These results indicate that *P. indica* can enhance soybean’s adaptive ability to saline-alkaline stress by regulating ROS scavenging capacity, osmotic adjustment substance content, and photosynthetic capacity, thereby promoting plant growth. This suggests that *P. indica* has great potential in improving soybean productivity in natural saline-alkaline soils.

## 1 Introduction

Soil salinization is a global ecological issue, with approximately 9.32×10^8^ hectares of land worldwide affected by this phenomenon (Negacz et al., 2022). The area of saline-alkaline soils globally is increasing at a rate of 10% annually (Abiala et al., 2018). Soil salinization not only leads to a reduction in arable land and a decrease in crop yields, but also adversely impacts the activity of functional microorganisms, biodiversity, ecosystem functions, and ecosystem services (Yu et al., 2018; Yang and Sun, 2020; Liu et al., 2024). At present, the Northeastern soda-saline land, one of the world’s three major soda-saline lands, covers an area of up to 3.96×10^7^ hectares (Zhao et al., 2020). The salinity in soda-saline soils is primarily composed of sodium carbonate (Na_2_CO_3_) and sodium bicarbonate (NaHCO_3_). These salts accumulate in the topsoil layer under the influence of transpiration, leading to poor soil aeration and nutrient immobilization, making these lands difficult to develop and utilize (Wang et al., 2017). Only a portion of the mildly saline-alkaline soils can be cultivated, while the cultivation of crops on moderately saline-alkaline soils may result in plant wilting, poisoning, or even root rot and death (Wang et al., 2024). However, as a reserve arable land resource, the development and utilization of moderately saline-alkaline soils is of great importance for increasing the total food production.

Plants grown in soda saline-alkaline lands face the combined stresses of salt stress and alkali stress. Salt stress primarily arises from an excess of cations. On the one hand, the large amounts of sodium ions in the soil compete with other ions, affecting mineral absorption, disrupting the ionic balance in root cells, and leading to plant water stress and physiological drought (Gogna et al., 2020). To reduce their own water consumption, plants will decrease transpiration and stomatal conductance, leading to a decline in photosynthetic rate (Zahra et al., 2022; Balasubramaniam et al., 2023). On the other hand, excessive salt ions can also increase the levels of reactive oxygen species and malondialdehyde in crops, alter cell membrane permeability, and affect normal cellular activities, thereby influencing plant growth and development (Hnilickova et al., 2021; Tomar et al., 2021). Alkali stress primarily originates from Na_2_CO_3_ and NaHCO_3_, which further increase soil pH on top of salt stress. Therefore, in addition to osmotic stress and ionic toxicity, the high pH can severely disturb the extracellular pH and affect the intracellular environment, damaging cell membrane integrity and organelle function (Ye et al., 2019). Furthermore, high pH can also induce plants to produce ethylene, inhibiting root development and impeding plant growth (Li et al., 2015). Overall, soil salinization and alkalinization have a significant impact on the absorption and metabolic functions of plants, seriously hindering agricultural production.

Currently, enhancing plant stress resistance and mitigating the damage caused by stress through microbe-plant symbiosis is an extremely effective approach (Singh et al., 2023; Ur Rahman et al., 2023). *P. indica* is a plant-symbiotic fungus that shares functional similarities with arbuscular mycorrhizal fungi. It has a broader host range and has been shown to play a role in enhancing plant disease resistance, growth promotion, and adaptation to environmental stresses (Li et al., 2022, 2023; Scholz et al., 2023). Mani et al. (2023) discovered that under water stress, *P. indica* can promote the development of lateral roots, increase root surface area, aid in nutrient absorption, and improve water use efficiency. Ghorbani et al. (2021) under heavy metal stress conditions with *P. indica* inoculation, found that *P. indica* can alleviate plant damage by enhancing the host’s photosynthetic rate and the content of soluble proteins and proline, thereby regulating osmotic balance. Zhang et al. (2022) found that *P. indica* can increase the activity of antioxidant enzymes such as superoxide dismutase (SOD) in soybeans under 200 mmol/L NaCl stress, reducing the accumulation of reactive oxygen species and mitigating cellular membrane damage. Based on these findings, we speculate that *P. indica* may hold considerable potential for alleviating the detrimental effects of soda-saline-alkaline stress on soybeans.

Soybean is a critically important economic and oil crop, with widespread applications in the food and industrial sectors. Soybean yield is of paramount significance for food security and agricultural economic development. In recent years, research into improving soybean salinity-alkali tolerance has gradually advanced, such as through the use of exogenous substances and genetic regulation (Feng et al., 2021; Fu et al., 2023; Zhang et al., 2023a). However, the effects of *P. indica* on the growth of soybean under natural soda saline-alkaline soil conditions remain unclear. Therefore, this experiment established a symbiotic system between *P. indica* and soybean under non-sterilized soda saline-alkaline soil conditions with two different levels of salinization, aiming to elucidate the influence of *P. indica* on soybean biomass, antioxidant enzyme activities, osmotic adjustment substances, and photosynthetic capacity. Based on the research background, we hypothesize that *P. indica* can enhance soybean tolerance to saline-alkaline stress and promote its growth by increasing antioxidant enzyme activities, osmotic adjustment substances, and photosynthetic capacity. The results of this study are expected to provide a theoretical basis for further understanding the mechanisms by which *P. indica* alleviates saline-alkaline stress, and offer new insights for increasing the utilization of soda saline-alkaline lands and improving soybean productivity in moderately saline-alkaline soils.

## 2 Materials and methods

### 2.1 Materials

*Piriformospora indica* (*P. indica*, provided by Prof. Wu Chu, College of Horticulture and Landscape, Yangtze University, China) was cultured to a concentration of 2×10^5^ CFU/mL using modified KM liquid medium at 28°C at 120 rpm (Hill and Kafer, 2001). The inoculant consists of culture fluid and mycelial mass. When inoculating, use a low-speed mixer to break up the mycelial mass and mix it with the culture solution. The fungus was expanded and preserved by the Key Laboratory of Coldland Ecological Restoration and Resource Utilization of Heilongjiang University.

Soybean seeds were selected as Hefeng 55, one of the main saline-alkaline tolerant soybean varieties grown in saline-alkaline farmland in Northeast China, purchased from Heilongjiang Provincial Academy of Agricultural Sciences. Soybean fertilizer was a compound fertilizer with the main components of N-P_2_O_5_-K_2_O, total nutrients≥40%, purchased from Heilongjiang Qingdong Sunshine Agricultural Bio-technology Co.

The test soil was taken from the soda saline-alkaline wasteland in Shangjia Village, Zhaodong City, Heilongjiang Province (E:125°89′, N:46°12′), and was divided into two kinds of saline-alkaline soils: mildly saline-alkaline soil (L-S, pH 8.47, EC 3.64 ds/m, salt content of 0.24%), moderately saline-alkaline soil (M-S, pH 9.28, EC 5.71 ds/m, salt content of 0.46%). The two soils were mixed separately after removing plant debris and soil bulks, and packed into plastic pots with specifications of 32 cm × 15 cm × 20 cm, each containing 3.5 kg of soil.

### 2.2 Experimental design

The experiment was conducted in June 2023 in a greenhouse on the Hulan campus of Heilongjiang University (E:126°59′, N:45°89′) using two-factor variational design with (1) soil salinity-alkalinity levels: mildly saline-alkaline soil (L-S), moderately saline-alkaline soil (M-S), (2) planting method treatment groups: no inoculation (NM), *P. indica* (Pi), and soybean composite fertilizer (F).

No inoculation treatment: 10 selected, healthy seeds were evenly scattered into each pot. They were covered with a 2 cm layer of soil.

Inoculation treatment: 10 selected, healthy seeds were evenly scattered into each pot. Concurrently, a 20 mL mixture of *P. indica* mycelium and culture solution, which had been cultured to a concentration of 2×10^5^ CFU/mL, was uniformly applied to the seeds and surrounding soil. Finally, the seeds were covered with a 2 cm layer of soil.

Fertilizer treatment: 2 g of fertilizer were spread at the bottom of each planting pot. Saline-alkaline soil was then added to the pots. 10 selected, healthy seeds were evenly scattered into each pot. They were covered with a 2 cm layer of soil.

A total of six treatments were established, each with five replicates, resulting in a total of 30 pots arranged in a randomized complete block design. Each pot was evenly sown with 10 pre-germinated seeds. After 10 days, the seedlings were thinned, leaving five plants per pot. The experiment was conducted under natural light conditions, with temperatures ranging from 20°C to 35°C. Watering was performed twice weekly, and samples were collected 50 days after planting for the determination of various indices.

### 2.3 Method

#### 2.3.1 Determination of soybean mycorrhizal colonization rate

Five samples were randomly selected for each treatment, cut 60-80 fresh soybean root segments of about 1 cm, and the root segments were stained by trypan blue staining (Phillips and Hayman, 1970). We have made minor modifications to the previously described method. In brief, the root segments were immersed in a 10% potassium hydroxide (KOH) solution and heated in a water bath at 90°C for 10 minutes. After rinsing with water, the segments were treated with a 5% hydrochloric acid (HCl) solution and acidified at room temperature for 5 minutes. Following another rinse, the root segments were stained with trypan blue solution and heated in a water bath at 90°C for 30 minutes. Finally, the stained samples were decolorized using a decolorizing solution. The stained root segments were observed under a 10x40 magnification microscope to assess the colonization. The colonization rate was calculated using the method described by Bierman and Linderman (Biermann and Linderman, 1981). The calculation formula is as follows:


$$\text{root colonization }(\%)=\frac{Number\;of\;root\;segments\;in\;which\;was\;observed\;}{Total\;number\;of\;segments\;studied}\times 100$$

#### 2.3.2 Determination of soybean biomass

For each treatment, take 5 soybean plants and clean the roots with distilled water, then clean them with deionized water three times, use filter paper to dry the surface moisture, and measure the fresh weight, shoot height, stem diameter, and root length of the whole plant.

#### 2.3.3 Determination of soybean malondialdehyde content and antioxidant enzyme activity

Malondialdehyde (MDA) was determined by thiobarbituric acid test (Zeng et al., 2022). Weigh 0.1 g of soybean leaves (m), grind it into homogenate in 1 mL of 5% trichloroacetic acid (TCA) solution, centrifuge it at 3000×g for 10 min, and take the supernatant to measure its volume (V). Take 400 μL of supernatant (V_1_) and mix it with 400 μL of 0.67% thiobarbituric acid (TBA), water bath at 100°C for 30 min, cool it down and centrifuge it again, and the supernatant is the solution to be measured (V_2_). The supernatant was taken and the absorbance values were measured at 450 nm, 532 nm and 600 nm, respectively, and the MDA concentration (C’) was calculated as follows:


$$C^{\prime }(\text{μmol}/\text{L})=6.45({\text{A}}_{532}-A_{600})-0.56A_{450}$$

The MDA content was calculated as follows:


$$MDA(\text{μmol}/\text{g})=\frac{C'\times V_{2}\times V}{m\times V_{1}\times 1000}$$

Superoxide dismutase (SOD) activity was determined by nitrogen blue tetrazolium method (Giannopolitis and Ries, 1977), with slight modifications. Weigh 0.1 g of soybean leaves (m), add 1 mL of 0.05 M pre-cooled phosphate buffer (pH=7.8) to grind into a homogenate, and centrifuge at 12,000×g for 15 min at 4°C, with the supernatant volume of V. Take 25 μL of supernatant (V_S_) and add color developer (750 μL 0.05 M phosphate buffer pH=7.8, 150 μL 130 mM methionine, 150 μL 750 μM NBT, 150 μL 100 μM EDTA-Na_2_, 150 μL 20 μM riboflavin, and 125 μL distilled water), and the control group add phosphate buffer instead of supernatant, and the tubes reacted for 20 min at 4000 lx sunlight for 20 min. Absorbance was measured at 560 nm, and SOD activity was calculated as follows:


$$SOD(U/g)=\frac{2(A_{ck}-A_{E})\times V}{A_{ck}\times m\times V_{s}}$$

Where A_ck_ is the control tube absorbance and A_E_ is the sample tube absorbance.

Catalase (CAT) activity was determined by ultraviolet spectrophotometry (Aebi, 1984). Weigh 0.1 g of soybean leaves (m), add 1 mL of extraction buffer (77 mg DTT, 5 g PVP, and volume to 100 mL with 0.1 M pH=7.5 phosphate buffer), grind to homogenate under ice-bath conditions, and centrifuge for 30 min at 12,000×g at 4°C, and volume of supernatant V. 100 μL of supernatant (V_S_) was taken and 2.9 mL of 20 mM H2O2 was added to the reaction system, and recording was started at 15 s. The absorbance value at 240 nm was taken as the initial value (OD_0_), and the absorbance value at 2 min was recorded (OD_2_). The formula for the calculation of CAT activity was as follows:


$$CAT(U/g/min)=\frac{(OD_{2}-OD_{0})\times V}{(t_{2}-t_{1})\times 0.01\times V_{S}\times m}$$

Where t_2_ is the reaction termination time and t_1_ is the reaction initial time.

Peroxidase (POD) activity was determined by guaiacol method (Cakmak and Marschner, 1992). Weigh 0.1 g of soybean leaves (m), add 1 mL of 0.1 M phosphoric acid (pH=7) extraction buffer, grind to homogenate under ice-bath conditions, centrifuge at 8000×g for 15 min at 4°C, and the volume of supernatant is V. 2 μL of appropriately diluted supernatant (V_S_) was added to 198 μL of reaction solution (100 mL of 0.1 M pH=6 phosphate buffer, 0.5 mL of guaiacol, and 1 mL of 30% H_2_O_2_ mixed thoroughly), and the absorbance at the beginning was measured at 470 nm (OD_0_), and the absorbance at the end of 3 min (OD_2_) was recorded. The formula for calculation of the POD activity was as follows:


$$POD(U/g/min)=\frac{(OD_{2}-OD_{0})\times V\times \text{N}}{(t_{2}-t_{1})\times m\times V_{S}}$$

Where t_2_ is the reaction termination time, t_1_ is the initial reaction time, N is the dilution factor.

#### 2.3.4 Determination of soybean osmotic adjustment substances content

Free proline (Pro) content was determined by the acidic ninhydrin colorimetric method (Borsai et al., 2020). Weigh 0.1 g of soybean leaves (m), add 1 mL of 3% sulfosalicylic acid solution to grind into a homogenate, boiling water bath for 10 min, centrifuged at 4000 × g for 10 min, and the supernatant liquid volume is V. Take 400 μL of supernatant, add 400 μL of glacial acetic acid and 400 μL of acid ninhydrin (1.25 g ninhydrin, 30 mL of glacial acetic acid, 20 mL of 6 M phosphoric acid, 70°C water bath mixed well) boiling water bath for 30 min, cool down and add 800 μL of toluene, shaking for 30 s, centrifuged at 3000 × g for 5 min, take the upper layer of the toluene solution at 520 nm to record absorbance values. The standard curve was calculated according to the above method using the standard proline, and the proline concentration of the leaves to be tested was calculated as C (μg/mL) according to the standard curve. The proline content was calculated as follows:


$$Pro(\text{μg}/g)=\frac{V\times C}{m}$$

Soluble sugar (SS) content was determined by the anthrone colorimetric method (Dien et al., 2019), with slight modifications. Weigh 0.1 g of soybean leaves (m), add 5 mL of distilled water and grind into homogenate, boil water bath for 30 min, filter the mixture to obtain the filtrate. Transfer the filtrate to a volumetric flask and adjust the volume to 25 mL with distilled water. Take 500 μL of filtrate after volume fixing, add 1.5 mL of distilled water, 0.5 mL of anthrone-ethyl acetate (1 g of anthrone, 50 mL of ethyl acetate was mixed thoroughly) and 5 mL of sulfuric acid, mix thoroughly and then immediately heat it in a boiling water bath for 1 minute, naturally cooled down to room temperature, and the absorbance value was recorded at 630 nm. The standard curve was calculated according to the above method using pure sucrose, and the concentration of soluble sugars in the leaves to be tested was calculated as C (μg/mL) based on the standard curve. The soluble sugar content was calculated as follows:


$$SS(mg/g)=\frac{C\times 7.5\times N}{m\times 1000}$$

Where 7.5 is the total volume of the reaction solution, N is the dilution factor, and 1000 is the conversion factor between μg and mg.

Soluble protein (SP) was determined by the Coomassie brilliant blue G-250 method (Wang and Huang, 2018). Weigh 0.1 g of soybean leaves (m_0_), add 5 mL of distilled water and grind it into homogenate, let it stand for 1 h at room temperature, centrifuge it at 4000×g for 20 min. Transfer the supernatant to a volumetric flask and adjust the volume to 10 mL with distilled water. Take 100 μL of the supernatant (V_S_) after volume fixing, add 5 mL of Coomassie brilliant blue G-250 solution (100 mg of Coomassie brilliant blue G-250 was dissolved in 50 mL of 95% ethanol, 100 mL of 85% phosphoric acid was added, and the volume was fixed to 1 L with distilled water), mix thoroughly, and then leave it to stand for 2 min, and then the absorbance value was recorded at 595 nm. The standard curve is calculated using Bovine Serum Albumin (BSA) as described above, and the soluble protein content of the liquid to be measured is calculated from the standard curve. The formula for calculating the soluble protein content is as follows:


$$SP(mg/g)=\frac{m\times 5.1\times N}{m_{0}\times V_{S}\times 1000}$$

Where 5.1 is the total volume of the reaction solution, N is the dilution factor, and 1000 is the conversion factor between μg and mg.

#### 2.3.5 Determination of soybean photosynthetic gas exchange parameters

Photosynthetic gas exchange parameters were determined using the LI-6400 photosynthesis meter (LI-COR Corporate, Lincoln, Nebraska, USA). On July 30, 2023 (50 days after soybean planting) from 9:00 AM to 11:00 AM, we selected plants with consistent growth in each treatment to measure the relevant indexes and selected the third leaf from the top in each treatment to measure three times. The indexes measured included net photosynthetic rate (*A*), stomatal conductance (*GH_2_O*), intercellular CO_2_ concentration (*Ci*), and transpiration rate (*E*).

#### 2.3.6 Statistical analysis

All data were processed and statistically analyzed using SPSS 25 (SPSS, Chicago, IL, USA). The data were checked to see whether they conformed to the normal distribution. If not, log conversion was performed. Significance of treatments was tested using one-way ANOVA and two-way ANOVA, and the significance of differences between groups was tested using the LSD test at the 0.05 level. Plotted using Origin2022 (Origin Lab-COR, Northampton, MA, USA). PCA (Biplot) and random forest plot were drawn using ggplot2 (Wickham, 2016), vegan (Oksanen et al., 2022), and rfPermute (Archer, 2023) in R (4.3.2) software.

## 3 Results

### 3.1 Effect of different treatment groups on mycorrhizal colonization rate of soybean

**Figure 1:**
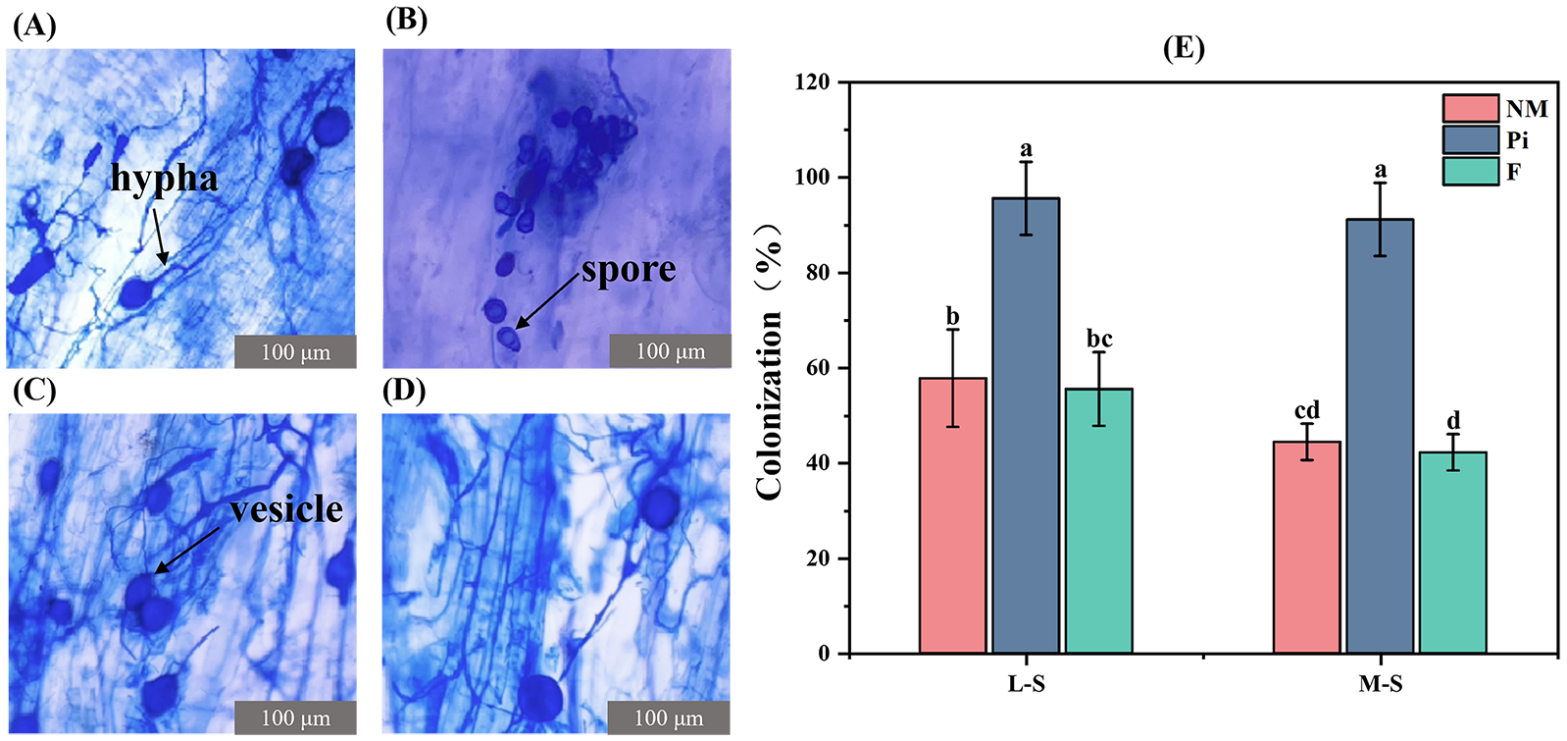
Colonization rate and colonization of soybean roots in each treatment group under the microscope (400 x). **(A)** AMF vesicle and mycelial structure in NM-treated group; **(B)** *P. indica* spore structure in Pi-treated group; **(C)** AMF vesicle and mycelial structure in Pi-treated group; **(D)** AMF vesicle and mycelial structure in F-treated group; and **(E)** soybean root colonization rate. Results are means ± standard deviation of five replicates. Different letters indicate differences between treatments (*P*<0.05).

*P. indica* established a good symbiotic relationship with the soybean root system. Clear *P. indica* mycelium and spore structures were observed in the root system of the Pi treatment group, while mycelium and vesicle structures of arbuscular mycorrhizal fungi (AMF) were also present (**Figures 1B, C**). The vesicle and mycelium structures of AMF were respectively observed in the NM and F treatment groups (**Figures 1A, D**), suggesting the presence of indigenous AMF in the natural saline-alkaline soil matrix. Root colonization rates were determined, and it was found that the increase in soil salinity-alkalinity level had no significant effect (*P*>0.05) on *P. indica* colonization (**Figure 1E**). The colonization rate did not differ significantly between the NM and F treatments, indicating that fertilizer application had no effect (*P*>0.05) on indigenous AMF colonization. The highest infestation rates of 95.6% and 91.1% were recorded in the Pi treatment groups under L-S and M-S conditions, respectively (**Figure 1E**), which were significantly different (*P*<0.05) from the other treatment groups.

### 3.2 Effect of different treatment groups on soybean biomass under saline-alkaline stress

**Figure 2:**
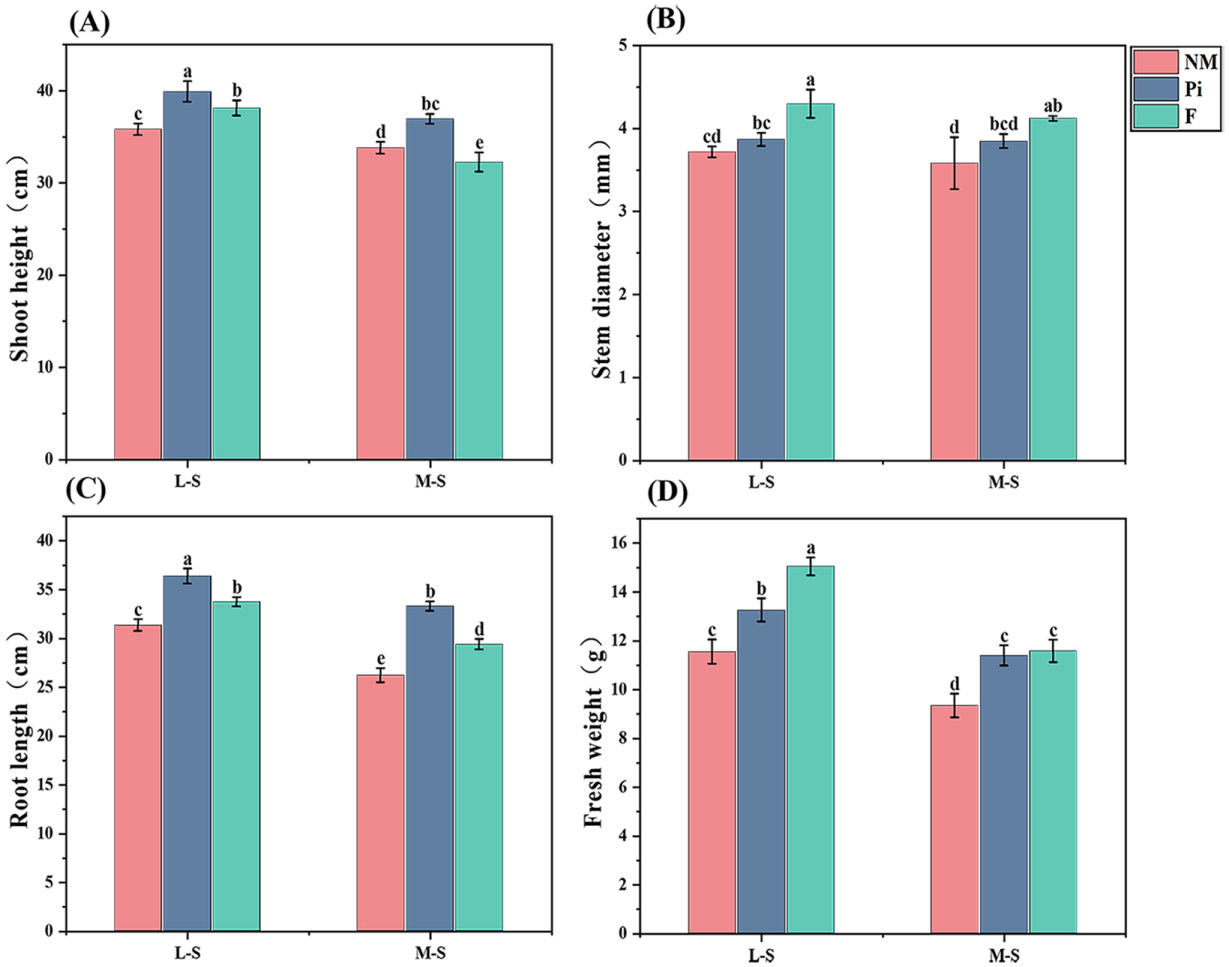
Growth parameters of soybean under different treatments. **(A)** Soybean shoot height; **(B)** Soybean stem diameter; **(C)** Soybean root length; **(D)** Soybean fresh weight. The results are the mean ± standard deviation of five replicates, and different letters indicate the differences between treatments (*P*<0.05).

The levels of soil salinity-alkalinity and the addition of fungi and fertilizers had highly significant (*P*<0.001) and interactive effects on soybean shoot height and root length (*P*<0.05) (**Table 1**). Shoot height and root length tended to decrease significantly with increasing soil salinity-alkalinity, and inoculation with *P. indica* increased soybean shoot height and root length more significantly (*P*<0.05) than the other treatments at both salinity-alkalinity levels (**Figures 2A, C**).Under M-S condition, shoot height and root length in the Pi treatment group increased by 9.3%, 14.6%, 27%, and 13.3% compared to the NM and F treatments, indicating that even under more severe saline-alkaline stress, *P. indica* can still alleviate the inhibition of soybean shoot height and promote root growth to a certain extent. However, the F treatment did not have a significant positive effect on soybean shoot height, although it did have a certain promotion effect on root development, which was significantly different from the Pi treatment.

**Table 1:** Results of two-way ANOVA on the effect of soil salinity-alkalinity levels and different treatments on the parameters measured in soybean.

Parameter measured	Soil salinity-alkalinity levels	Treatment	Soil salinity-alkalinity levels × Treatment
Fresh weight	137.294 ***	61.314 ***	5.175 *
Shoot height	207.286 ***	144.601 ***	4.231 *
Stem diameter	19.438 ***	49.096 ***	1.010 NS
Root length	83.240 ***	33.574 ***	8.565 **
MDA	1671.078 ***	709.470 ***	30.574 ***
SOD	1381.591 ***	144.444 ***	19.580 ***
POD	809.678 ***	505.007 ***	23.672 ***
CAT	33.333 ***	55.750 ***	4.083 *
Pro	1675.759 ***	1128.945 ***	5.274 *
SS	1084.783 ***	766.243 ***	149.769 ***
SP	76.914 ***	37.251 ***	4.937 *
*A*	217.549 ***	179.289 ***	22.133 ***
*Ci*	89.723 ***	90.264 ***	9.677 **
*E*	128.647 ***	86.843 ***	19.471 ***
*GH_2_O*	115.790 ***	95.997 ***	8.666 **

The numbers in the table represent the F-values obtained from the two-way ANOVA results. MDA, Malondialdehyde; SOD, Superoxide dismutase; POD, Peroxidase; CAT, Catalase; Pro, Proline; SS, Soluble sugars; SP, Soluble protein; *A*, Net photosynthetic rate; *E*, Transpiration rate; Ci, Intercellular CO2 concentration; *GH_2_O*, Stomatal conductance.

NS, not significant. *P < 0.05. **P < 0.01. ***P < 0.001.

Soil salinity-alkalinity levels with fungi and fertilizer additions had highly significant (*P*<0.001) effects on soybean stem diameter, but the interaction between both was not significant (*P*>0.05) (**Table 1**). Under L-S conditions, the F treatment had a significantly higher (*P*<0.05) stem diameter than the other treatment groups, and there was no difference (*P*>0.05) between the Pi and NM treatments (**Figure 2B**). With the increase of soil salinity-alkalinity, there were no significant differences (*P*>0.05) in stem diameter among the Pi, NM, and F treatments.

The determination of soybean growth indicators in different treatment groups showed that both soil salinity-alkalinity levels and the additions of fungi and fertilizers had highly significant (*P*<0.001) effects on soybean fresh weight, and there was an interaction between the two (*P*<0.05) (**Table 1**). Under L-S conditions, both F and Pi treatments significantly increased soybean fresh weight and were significantly different (*P*<0.05) from NM (**Figure 2A**). With the increase of soil salinity-alkalinity, soybean fresh weight decreased significantly (*P*<0.05). Under M-S condition, soybean fresh weight in the Pi and F treatments was still significantly higher than that in the NM treatment, while there was no significant difference between the Pi and F treatments. This proved that *P. indica* could still promote the accumulation of plant nutrients and the growth of plants even when the soil salinity-alkalinity was higher, and it achieved the same growth-promoting effect as the fertilization treatment.

### 3.3 Effects of different treatments on soybean MDA content and antioxidant enzyme activities in saline-alkaline environments

**Figure 3:**
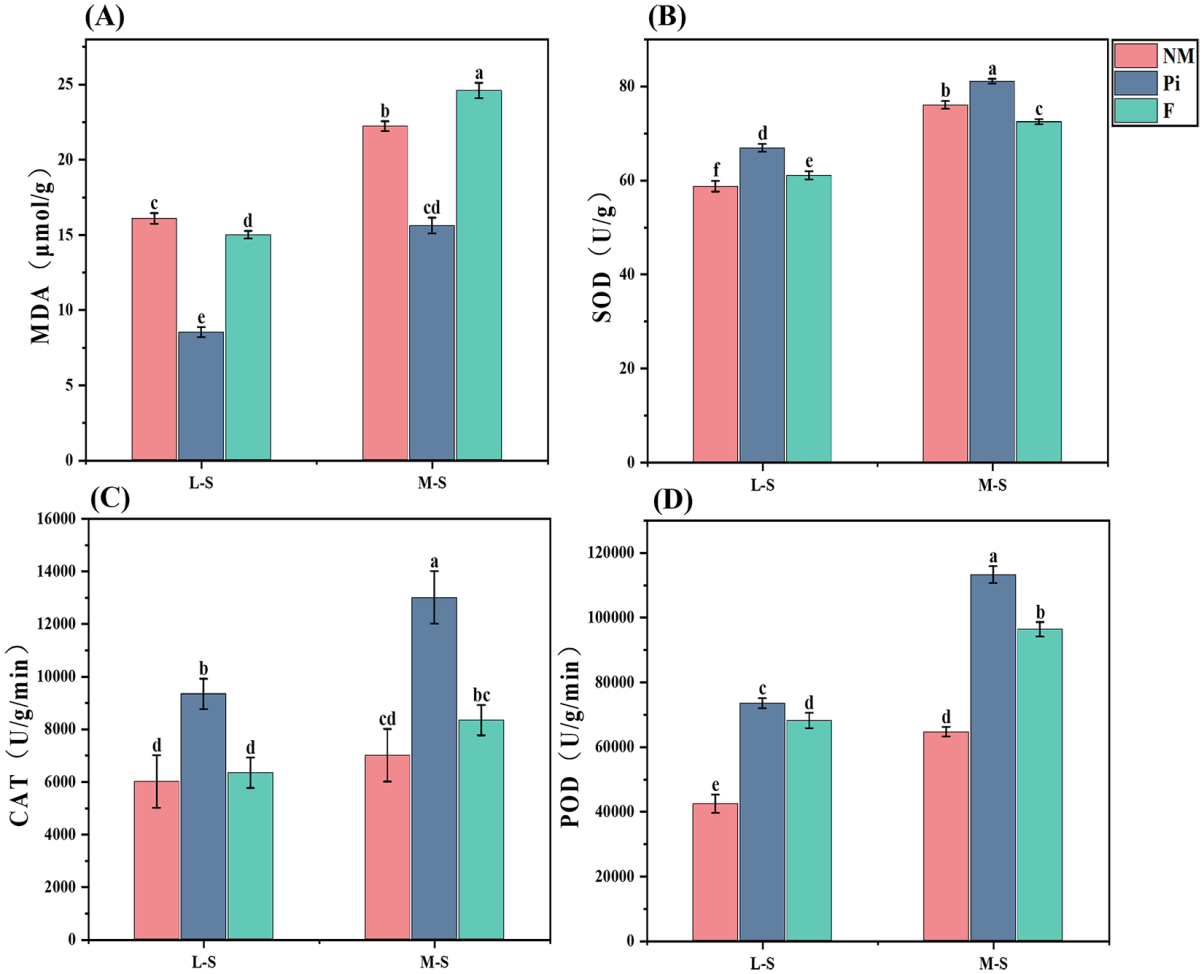
Antioxidant enzyme activities and MDA content of soybean leaves. **(A)** Malondialdehyde; **(B)** Superoxide dismutase; **(C)** Catalase; **(D)** Peroxidase. The results are the mean ± standard deviation of five replicates, and different letters indicate the differences between treatments (*P*<0.05).

Soil salinity-alkalinity levels and the additions of fungi and fertilizers had highly significant (*P*<0.001) effects on MDA content in soybean leaves, and there was a significant interaction between the two (*P*<0.05) (**Table 1**). Soybean plants exposed to saline-alkaline stress showed a progressive increase in MDA concentration as soil salinity-alkalinity deepened (**Figure 3A**). The addition of *P. indica* significantly reduced the MDA content of soybean leaves under salinity stress, and there was a significant difference between it and the other treatment groups (*P*<0.05). Under the M-S condition, the Pi treatment reduced MDA content by 29.8% and 36.6% compared to the NM and F treatments, respectively.

The analysis of soybean SOD, CAT, and POD activities in soils with different levels of salinity-alkalinity demonstrated (**Figures 3B–D**) that soil salinity-alkalinity levels and the addition of both fungi and fertilizers significantly affected the activities of the three antioxidant enzymes (*P*<0.001), and there was an interaction between the two (*P*<0.05) (**Table 1**). There was a gradual increase in the activities of SOD, CAT, and POD with the increase in stress levels. Under M-S conditions, the activities of the three antioxidant enzymes in the Pi-treated group were significantly higher than those in the treatment groups under L-S conditions, and there were significant differences between them and those in the NM and F-treated groups (*P*<0.05). The SOD activities increased by 6.6% and 11.9% in compared with those in the NM and F-treated groups; the CAT activities were 1.86 times and 1.56 times higher than those in the NM and F-treated groups, respectively. Inoculation with *P. indica* increased POD activities most significantly, followed by the F-treated groups, and there were significant differences between them and the NM-treated groups. The addition of *P. indica* helped soybeans resist saline-alkaline stress to a certain extent by increasing the activity of antioxidant enzymes and decreasing the MDA content in soybeans. The addition of soybean fertilizer only significantly enhanced POD activity. Fertilizer application in mildly saline-alkaline soils reduced soybean MDA content to a certain extent, whereas in soils with higher salinity-alkalinity, it significantly increased soybean MDA content.

### 3.4 Effects of different treatments on soybean osmotic adjustment substances under saline-alkaline stress

**Figure 4:**
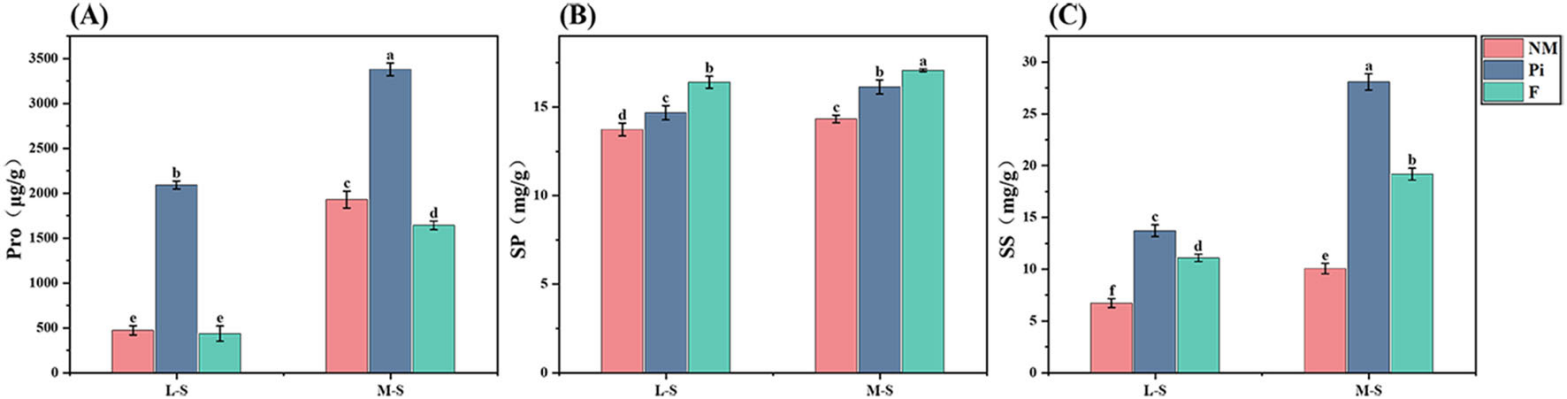
Content of osmotic adjustment substances in soybean leaves. **(A)** Proline; **(B)** Soluble Protein; **(C)** Soluble Sugars. The results are mean ± standard deviation of five replicates, and different letters indicate the differences between treatments (*P*<0.05).

Free proline, as an osmotic adjustment substance produced by plants themselves, plays an important role in regulating abiotic stresses. It was found that the levels of soil salinity-alkalinity and different treatments significantly affected the free proline (Pro) content of soybean leaves (**Table 1**). Saline-alkaline stress increased proline content in soybean, and the content increased significantly with deeper soil salinity-alkalinity (*P*<0.05) (**Figure 4A**). In both saline-alkaline soils, Pi treatment was able to increase the Pro content and was significantly different from the other two treatments (*P*<0.05). Under M-S condition, the proline content of the Pi treatment group was 1.75 and 2.06 times higher than that of the NM and F treatment groups, respectively.

Both soil salinity-alkalinity levels and different treatments had significant effects on soybean soluble protein (SP) content, and there was a significant interaction between them (*P*<0.05) (**Table 1**). With the deepening of soil salinity-alkalinity, the soluble protein content of soybean leaves in different treatment groups gradually increased (**Figure 4B**). In both saline-alkaline soils, the soluble protein content of the F treatment was significantly higher than that of the other treatment groups (*P*<0.05). The Pi treatment group also had a significantly positive effect on the increase of soluble protein content. Under the M-S condition, it increased by 12.6% compared to the NM treatment group.

Soil salinity-alkalinity levels and different treatments significantly affected the soluble sugar (SS) content of soybean (*P*<0.001) (**Table 1**). With the increase of soil salinity-alkalinity, the soluble sugar content of soybean leaves in different treatment groups increased significantly (**Figure 4C**). In both saline-alkaline soils, the soluble sugar content of the F group was significantly higher than that of the NM group, while the soluble sugar content of the Pi treatment group was significantly higher than that of the other two treatment groups (*P*<0.05). Under the M-S condition, the soluble sugar content of the Pi group was 179.7% and 46.5% higher than that of the NM and F groups, respectively.

### 3.5 Effect of different treatments on soybean photosynthetic gas exchange parameters under saline-alkaline stress

**Figure 5:**
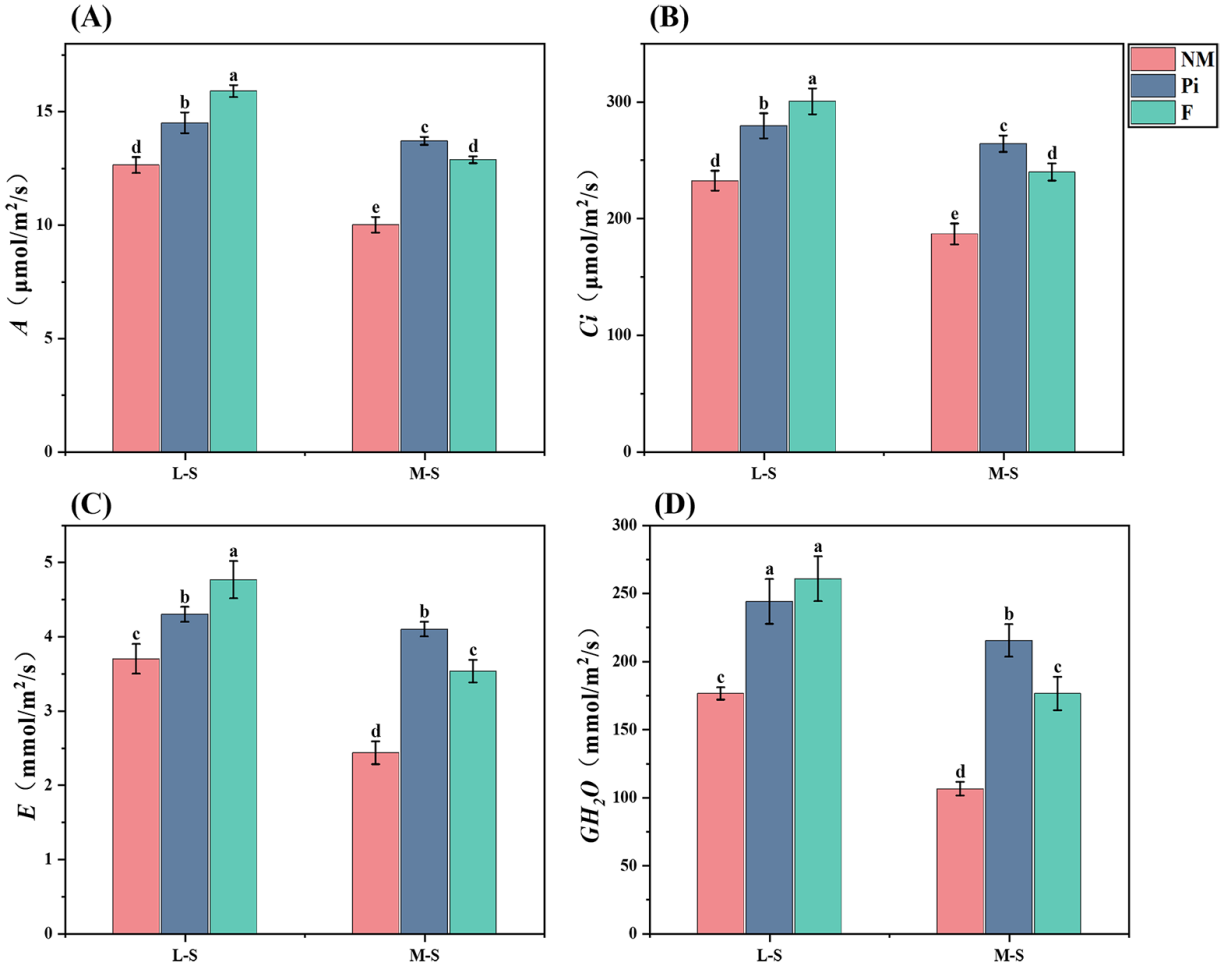
Photosynthetic gas exchange parameters of soybean leaves. **(A)** *A*: Net photosynthetic rate; **(B)** *Ci*: Intercellular CO_2_ concentration; **(C)** *E*: Transpiration rate; **(D)** *GH_2_O*: Stomatal conductance. results are means ± standard deviation of three replicates. Different letters indicate differences among treatments (*P*<0.05).

The net photosynthetic rate (*A*) of soybean leaves in soils with different levels of salinity-alkalinity was determined (**Figure 5A**) and it was found that both the level of soil salinity-alkalinity and the different treatments significantly (*P*<0.001) affected the net photosynthetic rate (**Table 1**). Under different levels of soil salinity-alkalinity, the net photosynthetic rate of soybean leaves was significantly higher (*P*<0.05) in both the Pi and F treatment groups than in the NM group. Under the M-S condition, *P. indica* showed the most significant increase in net photosynthetic rate.

Intercellular carbon dioxide concentration (*Ci*), transpiration rate (*E*), and stomatal conductance (*GH_2_O*) of soybean under different treatments were measured (**Figures 5B–D**). The results showed that the level of soil salinity-alkalinity and the different treatments significantly affected these indexes (*P*<0.001), and there was an interaction relationship (**Table 1**). Soybean *Ci*, *E*, and *GH_2_O* all showed a significant (*P*<0.05) decrease with increasing saline-alkaline stress. Under the M-S condition, all four photosynthetic gas exchange parameters (*A*, *E*, *Ci*, and *GH_2_O*) of soybeans in the Pi treatment group were significantly higher (*P*<0.05) than those in the NM and F treatment groups.

### 3.6 Principal component analysis and driving factors underlying changes in soybean biomass

**Figure 6:**
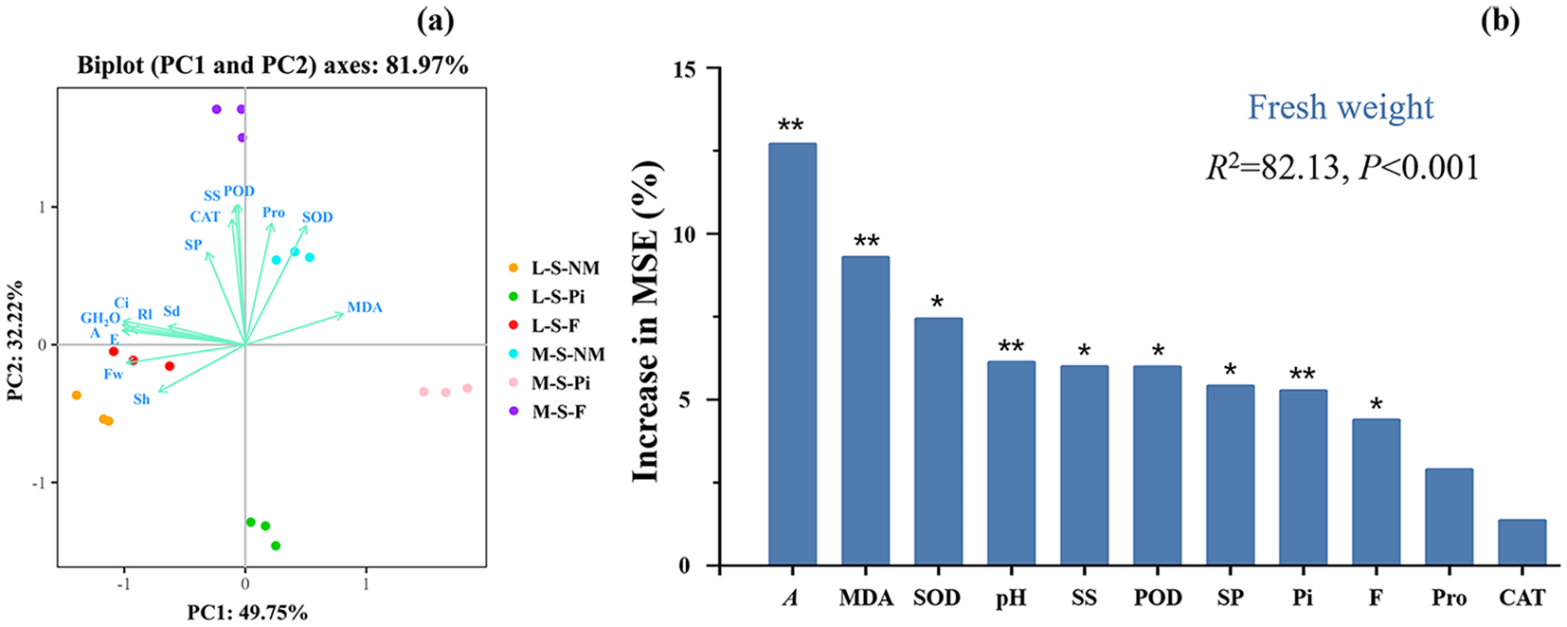
Principal component analysis of soybean physiological and biochemical indicators and drivers of soybean biomass change. Rl, Root length; Sh, Shoot height; Sd, Stem diameter; Fw, Fresh weight; A, Net photosynthetic rate; Ci:,Intercellular CO_2_ concentration; E, Transpiration rate; GH_2_O: Stomatal conductance; MDA, Malondialdehyde; SOD, Superoxide dismutase; POD, Peroxidase; CAT, Catalase; Pro, Proline; SS, Soluble sugars; SP, Soluble protein; F, Fertilizer; Pi, *P. indica*. *P < 0.05. **P < 0.01.

We analyzed the data of soybean growth indexes, antioxidant enzyme activities, osmotic adjustment substances, and light parameters as principal components, and the X and Y axes respectively indicated the first (PC1) and second (PC2) principal components (**Figure 6A**), with a total degree of explanation of 81.97%. The results showed that photosynthetic parameters and MDA content contributed more to PC1, while antioxidant enzyme activities and soybean growth indexes contributed more to PC2. MDA was negatively correlated with both photosynthetic parameters and soybean growth indexes, and positively correlated with antioxidant enzyme activities and osmoregulatory substances. In this analysis, the distinction between samples was obvious, indicating that the differences between treatments were more significant.

Random forest analysis of data on fungal and fertilizer treatments, antioxidant enzyme activities, osmotic adjustment substances, and net photosynthetic rate predicted the major physiological and biochemical factors affecting changes in soybean biomass in a natural soda salinity-stressed soil (**Figure 6B**). The results had a total explanatory value of 82.13%, with *A* being the most significant variable in predicting soybean biomass, followed by MDA, soil pH, and *P. indica*. In addition, fertilizers, POD, SOD, SP, and SS were likewise significant factors in predicting changes in soybean biomass.

## 4 Discussion

### 4.1 Soybean root colonization and biomass effects of different treatments

In this study, inoculation with the *P. indica* resulted in a high colonization rate of the soybean root system, indicating good compatibility between soybeans and *P. indica* in natural soda saline-alkaline soil. Additionally, in the presence of indigenous arbuscular mycorrhizal fungi (AMF), inoculation with *P. indica* significantly increased soybean biomass. This increase may be due to the synergistic effects of the indigenous AMF and *P. indica* under saline-alkaline stress conditions, which together enhanced soybean resistance and promoted growth (Zhang et al., 2023b). Previous research has shown that increased salt concentrations adversely affect the mycelial growth and spore formation of AMF, reducing the colonization rate (Bothe, 2012). Our results are in agreement with Bothe et al. (**Figure 1E**). However, the colonization rate in the *P. indica*–inoculated treatment did not exhibit a significant decrease. This may be because *P. indica* can produce indole-3-acetic acid (IAA) during the liquid culture process (Sirrenberg et al., 2007). The production of IAA promotes seed germination and root development, enabling *P. indica* spores to contact the root system and successfully colonize at an early stage, thereby mitigating the adverse effects of salt on the spores and hyphae.

Biomass is one of the crucial indicators for evaluating plant tolerance to saline-alkaline stress. Saline-alkaline stress can alter the extracellular pH and affect the intracellular environment, suppressing cell growth and limiting plant development, ultimately leading to a reduction in biomass (Kotula et al., 2020; Zhao et al., 2023). Additionally, the saline-alkaline environment reduces the vitality of plant roots, impeding root growth and reducing nutrient supply, which in turn impacts biomass accumulation (Zhang et al., 2017). This study found that as soil salinity-alkalinity increased, soybean plant height, root length, and biomass declined significantly. However, the addition of microbial inoculants effectively alleviated these deleterious effects. At varying levels of saline-alkaline stress, the addition of *P. indica* increased soybean plant height, root length, and biomass, and even under the M-S condition, the growth-promoting effect of Pi treatment on soybeans was not significantly different from that of fertilization treatments (**Figure 2D**). This suggests that *P. indica* treatment significantly improved soybean tolerance to saline-alkaline stress. Mani et al. (2023) found that under drought conditions, inoculation with *P. indica* promoted the development of lateral roots, increased root volume, and enhanced the water use efficiency of rice. Therefore, we speculate that the reason by which *P. indica* enhances soybean’s tolerance to saline-alkaline stress may be that *P. indica* promotes root system development and increases root surface area, thereby alleviating the physiological drought in soybeans, and helping the plant to better absorb nutrients and water, thus facilitating growth and mitigating the damage caused by saline-alkaline stress to the cells.

### 4.2 Soybean MDA content and antioxidant enzyme activities under saline-alkaline stress of different treatments

Saline-alkaline stress can lead to the excessive production and accumulation of reactive oxygen species (ROS) such as superoxide anions, hydrogen peroxide, and hydroxyl radicals within plant cells (Lu et al., 2023). Huang et al. (2019) reported that when ROS accumulate to high concentrations, they can cause oxidative damage to biological molecules such as cell membranes, proteins, and nucleic acids, resulting in the production of MDA. Therefore, plants must maintain a balance between the generation and elimination of these reactive oxygen species. In our experiment, under saline-alkaline stress conditions, the MDA content in soybeans increased, indicating that the saline-alkaline stress caused oxidative damage to soybean cells, and the degree of damage increased with the increasing salinity-alkalinity of the soil (**Figure 3A**). Previous research has demonstrated that under salt stress, the application of ammonium nitrate can increase the nitrogen content in leaves to a certain degree. However, when the level of salt stress is higher, the application of ammonium nitrate can actually lead to a decrease in plant biomass. The primary reason is that a large accumulation of salt ions in the leaves causes an imbalance in osmotic pressure, resulting in cellular oxidative damage (Larbi et al., 2020). In this study, under the lower level of saline-alkaline stress, the MDA content in the F treatment group was lower than that in the NM group, possibly because mineral fertilizers provided an abundance of nutrients, ensuring sufficient nutrient supply and enhancing the antioxidant defense capacity of soybeans. However, at the higher stress levels, the F treatment led to a significant increase in MDA content. This finding further corroborates the results reported by Larbi et al. (2020).

In this study, soybeans reduced the oxidative damage caused by saline-alkaline stress by enhancing the activities of antioxidant enzymes. As the salinity-alkalinity of the soil increased, the activities of SOD, POD, CAT in soybeans all increased (**Figures 3B–D**). Within the plants, SOD is mainly responsible for converting the highly oxidizing superoxide anion into the more stable hydrogen peroxide, while POD and CAT are responsible for the further conversion of hydrogen peroxide into water and oxygen (Alscher et al., 2002; Kavian and Safarzadeh, 2022).The synergistic action of these three enzymes, as part of the plant’s antioxidant defense system, alleviated the oxidative damage to plant cells caused by saline-alkaline stress and protected the integrity of cellular structures and functions (Sadak et al., 2023).In this experiment, the soybeans in the *P. indica* addition group had higher SOD, POD, and CAT activities than those in the NM and F groups, indicating that *P. indica* accelerated the rate of removal of reactive oxygen species, which may be the reason for the significantly lower MDA content in the Pi treatment group compared to the other treatment groups. Song et al. (2019) reported that the application of an appropriate amount of nitrogen fertilizer under salt stress could alleviate the negative effects by enhancing the photosynthesis of plants. In our study, the POD activity in the fertilization treatment group was significantly higher than that in the NM group. This may also be related to the nitrogen in the fertilizer. Under saline-alkaline stress conditions, nitrogen augments the activity of antioxidant enzymes by enhancing the plant’s nutrient uptake and photosynthetic capacity. It appears that *P. indica* significantly outperforms other treatment groups in enhancing the antioxidant defense system of soybeans under soda saline-alkaline stress and can significantly alleviate the damage caused by saline-alkaline stress.

### 4.3 Soybean osmotic adjustment substances content under saline-alkaline stress of different treatments

In saline-alkaline soils, the presence of high concentrations of inorganic salt ions results in a soil osmotic pressure that exceeds the osmotic pressure within plant cells, affecting the ability of plant roots to absorb water. Concurrently, a large amount of Na^+^ competes with other ions, leading to an excessive accumulation of Na^+^ within plant cells, which disrupts the membrane structures and protein structures, resulting in an imbalance in osmotic pressure and physiological drought (Richards, 1947). To counteract these effects, plants accumulate osmotic adjustment substances, such as free proline, soluble sugars, and other compounds, to maintain normal cellular osmotic pressure, protect the integrity of membranes and proteins, and ensure the normal absorption and utilization of water by the plant (Couée et al., 2006; Zulfiqar and Ashraf, 2023). In our study, the contents of Pro, SS, and SP in soybean cells increased with the rising salinity-alkalinity of the soil (**Figures 4A–C**), indicating that the plants had activated their own defense systems to resist the osmotic imbalance caused by saline-alkaline stress, but with limited effect. However, Pi treatment significantly increased the contents of Pro, SS, and SP in soybean, indicating that under saline-alkaline stress conditions, *P. indica* can induce the accumulation of osmotic adjustment substances in soybean, reduce cellular osmotic potential, and enhance soybean’s tolerance to saline-alkaline stress. Similar results have been reported by Li et al. in their study on the alleviation of salt stress in alfalfa by *P. indica* (Li et al., 2017). Previous research has found that under stress conditions, the application of NPK fertilizers can promote the production of SP and reduce the synthesis of Pro (Wang et al., 2020). In this experiment, the SP content increased while the Pro content decreased in the F treatment group, consistent with the results of Wang et al. (2020). The possible reason is that the inorganic fertilizer provided the necessary nutrients for soybeans, helping them adapt better to environmental stress and reducing the need for proline synthesis within the plant.

### 4.4 Soybean photosynthetic gas exchange parameters under saline-alkaline stress of different treatments

As a key physiological mechanism for plant energy conversion and substance synthesis, photosynthesis is constrained by various environmental factors. Under soda saline-alkaline stress, the excessively high ion concentrations and pH values in the soil can damage cellular structures such as cell membranes, thereby impairing photosynthetic organs like chloroplasts, altering the intracellular environment, and consequently exerting adverse effects on the plant’s photosynthetic capacity (Zhang et al., 2023c). Previous studies have shown that inoculation with *P. indica* can enhance the photosynthetic rate of plants (Moreira et al., 2015) (Ghorbani et al., 2018). also found that under salt stress, the photosynthetic gas exchange parameters of tomato plants treated with *P. indica* were significantly higher than the uninoculated controls. Our experiment yielded similar results. In this study, under moderately soda saline-alkaline soil conditions, the soybean leaves in the Pi treatment group exhibited higher values of *A*, *E*, *Ci*, and *GH_2_O* compared to the NM and F groups (**Figures 5A–D**). This may be because the inoculated soybean enhanced their CO_2_ assimilation rate to improve photosynthetic capacity, thereby mitigating the damage caused by saline-alkaline stress (Al-Absi and Al-Ameiri, 2015). The improved photosynthetic capacity may also account for the increased accumulation of osmotic adjustment substances in the Pi treatment group. Under moderate saline-alkaline stress, the F treatment showed significantly lower photosynthetic parameters than the Pi treatment, which may be due to the high soil electrical conductivity leading to an imbalance in cellular osmotic pressure and damage to the photosynthetic organs. These results indicate that inoculation with *P. indica* can significantly improve the light energy utilization efficiency of soybeans under soda saline-alkaline stress, effectively enhancing the nutrient accumulation and salt-alkali tolerance of soybeans.

## 5 Conclusion

Under mild to moderate soda saline-alkaline stress conditions, inoculation with *P. indica* was found to significantly alleviate the detrimental effects on soybean. Specifically, *P. indica* inoculation enhanced the antioxidant enzyme activities in soybean leaves, promoted photosynthetic capacity, and increased the accumulation of osmotic adjustment substances. In sum, these physiological improvements led to increased soybean biomass. The findings from our study will provide a theoretical basis for improving soybean yields in soda saline soils in the future.

## Data Availability

The raw data supporting the conclusions of this article will be made available by the authors, without undue reservation.
